# Visibility & support for first generation college graduates in medicine

**DOI:** 10.1080/10872981.2021.2011605

**Published:** 2022-01-02

**Authors:** Abraham Gallegos, Lynn K. Gordon, Gerardo Moreno, Sue Nahm, Kathleen Brown, Valencia Walker, Vanessa Rangel, Stephanie Clavijo, Alejandra Casillas

**Affiliations:** a Department of Pediatrics, David Geffen School of Medicine at UCLA, Los Angeles, CA, USA; b Department of Ophthalmology, David Geffen School of Medicine at UCLA, Los Angeles, CA, USA; c Department of Family Medicine, David Geffen School of Medicine at UCLA, Los Angeles, CA, USA; d David Geffen School of Medicine at UCLA, Los Angeles, CA, USA; e Department of Radiological Sciences, David Geffen School of Medicine at UCLA, Los Angeles, CA, USA; f Division of Neonatology, Department of Pediatrics, David Geffen School of Medicine at UCLA, Los Angeles, CA, USA; g UCLA Fielding School of Public Health, Charles R. Drew University of Medicine and Science, Los Angeles, CA, USA; h Division of General Internal Medicine and Health Services Research, Department of Medicine, David Geffen School of Medicine at UCLA, Los Angeles, CA, USA

**Keywords:** First generation, medical student, medical school program, underrepresented minority

## Abstract

Of Being a First Generation (First Gen) college graduate is an important intersectionality which impacts the lens through which First Gen students learn to become physicians. In this Perspective, we define the First Gen identity and review some of the salient First Gen literature as it applies to the medical school experience. We discuss the conception, design and execution of First Gen initiatives and program development at our medical school as a call to action and model for other institutions to create communities for their First Gen populations, focusing on inclusion and tailored support. We describe the framework through which we envisioned our programming for First Gen medical students, trainees, staff, and faculty at the David Geffen School of Medicine at UCLA.

## Introduction

First Gen students are those whose parents did not attend college or obtain a college degree [1–8]. They are an important population in academia given the unique experiences and challenges they must overcome. Undergraduate universities across the USA have launched educational and administrative initiatives to support these students [9,10]. Some of these initiatives focus on university culture readiness, financial wellness, stereotype threat, imposter syndrome, psychological family stressors and lack of professional-social networks [11–15]. Unfortunately, there is a lack of similar programming in medical education.

First Gen students matriculating into medical school encounter difficult decisions about their future (e.g., choosing a medical specialty or career path) without the input of college-educated adults in their proximate relationships. Additionally, First Gen students suffer from a higher level of stress, physical and emotional fatigue, lower perceived social support and are less likely to practice self-care [16,17]. First Gen students are more likely to come from an underrepresented minority group and lower socioeconomic background [18–20]. Medical education is beginning to recognize First Gen students as a unique group of students. In 2017, the American Medical College Application Service (AMCAS), included a new First Gen indicator within the application system[21]. Most recently, the American Association of Medical Colleges’ (AAMC) developed a First Gen work group to create an on-line toolkit with resources for students and their advisors [22,23]. It is important this increased recognition be followed by the development of First Gen medical school programs. This manuscript highlights the development of a First Gen program at our medical school and, to our knowledge, is the first of its kind.

## Approach

The process of developing and implementing the early stages of our First Gen program relied on capitalizing on a university level First Gen initiative, working collaboratively with similar programs and receiving input from First Gen students.

A university system-wide First Gen visibility campaign was launched in 2017 and medical school faculty at our campus became involved[24]. These faculty collaborated with the undergraduate program prior to approaching the medical school leadership about developing a First Gen program at the school of medicine. A series of early meetings including First Gen medical students were held to gauge interest, share experiences and highlight unmet needs. These meetings mobilized a core group of students who then developed a mission statement charter, proposed key action items for medical school leadership and selected a faculty advisor. This First Gen organizational charter is available upon request. A First Gen listserv was thus created and further student feedback was obtained related to program components and future events. The First Gen program at the school of medicine was officially created in 2018.

## Results

A detailed description of our First Gen programming can be found in Table 1. Our programming consists of four focus areas: Community Building, Mentorship, Educational Transitions & Home Identity and Academic Support.

**Table 1.** First gen program components at our medical school

Program Component	Description *First Gen Focus Area(s)*	Duration
First Gen Medical School Organization	Student-led programming and events on career-building topics with a focus on the intersectionality with being First Gen. *Mentorship* *Educational Transitions & Home Identity* *Academic Support*	Longitudinal
First Gen Medical Visibility	First Gen lapel pin (student designed) that First Gen students, trainees, and physician faculty wear on their white coats. *Community Building*	Longitudinal
Academic Support Partnership	Academic Support resources and programming better aligned to meet the needs of the diverse population of students. *Community Building* *Mentorship* *Academic Support*	Longitudinal
First Gen Families	First Gen medical students, trainees, staff and faculty meet as a unit/family several times throughout the year in informal settings, to share/mentor and get to know one another. *Community Building* *Mentorship*	Longitudinal
White Coat Ceremony Family Welcome	Luncheon for parents, guardians, and other family members of matriculating First Gen medical students occurring just prior to the school’s White Coat Ceremony. *Community Building* *Educational Transitions & Home Identity*	Orientation
Community Dinner	First Gen students, residents, fellows, staff, and faculty come together to welcome the incoming class. The evening involves introductions by various student diversity group leaders, deans from the Office of EDI, and a First Gen keynote speaker. Additionally, organizers facilitate breakout sessions to promote socialization and discussions about First Gen experiences. Hearing stories from First Gen faculty provides an empowering and meaningful experience for the First Gen learners. *Community Building* *Mentorship*	Orientation
First Gen Welcome Soiree	First Gen medical students are invited to attend the First Gen Welcome Soiree hosted by the university in October, where all First Gen students from the entire campus come together. Medical students have typically engaged and networked with pre-medical First Gen undergraduate students at this event for the last two years. *Community Building* *Mentorship*	Fall
Medical School Graduation	First Gen graduating medical students received visible recognition during the commencement ceremony. They wear blue and gold cords over the graduation robe, representing their First Gen status. *Community Building* *Mentorship*	Spring

ß

1) Community Building: Visibility and Recognition

First Gen status is sometimes described as a ‘hidden identity.’ This sentiment was echoed during early planning sessions with a point of emphasis made in regards to meeting other First Gen peers and faculty/mentors. Our program focuses on increasing First Gen visibility and community at multiple different levels from matriculation to graduation. A salient example of this is the First Gen Community Dinner during orientation week and the First Gen robe cords that students wear at graduation (Table 1).

2) Mentorship: Intersectionality and Intergenerational

Our program provides mentorship at various stages of the training and career timeline via our First Gen Families component. Each ‘family’ is comprised of a MD/PhD faculty ‘family head,’ at least one resident/fellow trainee, 3–5 medical students, and First Gen staff members. These families foster mentorship among all levels of the career development pathway and create close-knit First Gen support systems.

3) Educational Transitions & Home Identity

First Gen students receive less informational and financial support from their family, and commonly assume a supportive role in their family [25,26]. Families wanting to better support their First Gen student may not know effective ways to do so. Our First Gen program aims to increase family engagement and understanding, as well as provide family members tangible ways to provide support. We host a luncheon for family members of matriculating students. Families are welcomed by medical school leadership, provided a presentation covering the timeline of medical school and the road to becoming a physician (including licensing and board exams, applying to residency and entering professional careers) and current First Gen students share examples of ways family members can support their First Gen student.

4) Academic Support that is First Gen focused

Our First Gen organization has partnered with the Director for Academic Student Support in the Dean’s office to strengthen academic support of First Gen students. We emphasize providing support at key points of transition, including: transitioning into medical school, preparation for the USA Medical Licensing Examination (USMLE) Step 1, core clinical clerkship experiences and the accompanying clerkship shelf exams. We also emphasize making the ‘hidden curriculum’ less hidden as it is an area where First Gen students can struggle to navigate[27]. The ‘hidden curriculum’ refers to experiences, resources and effective methods of studying that are considered common knowledge among many students, but are not explicitly taught in medical institutions[28].

The Academic Support office provides enhanced support for First Gen students via:

- Opportunities for First Gen faculty and students to self-identify on questionnaires and tutor/mentor request forms; to then deploy these mentors to reach out to First Gen students during key transition times
- Visibility of Academic Support staff at all First Gen meetings
- Promotion of Academic Support resources
- Special allotted meetings with Academic Support early in the school year to become familiar with learning resources and build connection to Academic Support staff.

## Discussion: next steps and lessons learned

Raising awareness of the First Gen experience in medical education is an important step in cultivating an academic culture of recognition, support and inclusion. We outline the key aspects of our approach in the development and implementation of a First Gen program at our medical school. Our approach demonstrates how we started with a First Gen student voice, then capitalized and used existing infrastructure to propel First Gen issues, tailored existing tools to build a First Gen program, and approached administration strategically with incremental asks: specifically by starting ‘simple’ and then building out as the perception for First Gen specific programming gained traction across the medical school. Our program broadens our First Gen focus to also include residents, fellows, faculty, staff, and alumni who identify as First Gen, creating a more effective network. At our school, we have used the Dean’s office for Equity, Diversity, and Inclusion as a central access point for our First Gen efforts. Through this office, we are connecting with leaders and champions for First Gen support in each of the respective departments and divisions, sowing the seeds for an enduring First Gen medical network.

We encourage other medical school programs to consider implementing their own First Gen program at their medical institution. Our role as medical leaders and educators – is to maximize the full potential of the First Gen medical student population. This population is poised to already understand and eliminate the structural injustices in medicine that lend themselves to innumerable health disparities and poor quality of health care, especially for the most marginalized and underserved populations. When we focus on cultivating our First Gen medical students, trainees, faculty and staff – we are taking steps to improve the future state of healthcare delivery for all our patients.
